# Association between maternal decision-making and mental health and the nutritional status of children under 6 years of age in sub-urban Nigeria

**DOI:** 10.1186/s12889-023-16055-2

**Published:** 2023-06-15

**Authors:** Morenike Oluwatoyin Folayan, Ayodeji Babatunde Oginni, Maha El Tantawi, Abiola Adeniyi, Michael Alade, Tracy L Finlayson

**Affiliations:** 1 Obafemi Awolowo University, Ile-Ife, Osun State Nigeria; 2 Innovative Aid, Abuja, Nigeria; 3 Faculty of Dentistry, Alexandria University, Alexandria, Egypt; 4 Lagos State University College of Medicine, Ikeja, Lagos State Nigeria; 5 Obafemi Awolowo University Teaching Hospitals’ Complex, Ile-Ife, Osun State Nigeria; 6 School of Public Health, San Diego State University, San Diego, CA USA

**Keywords:** Thinness, Stunting, Underweight, Overweight, Self-efficacy, Nigeria

## Abstract

**Background:**

We assessed the association between decision-making power and mental health status of mothers and the nutritional status of their children less than 6 years old in Ile-Ife, Nigeria.

**Methods:**

This was a secondary data analysis of 1549 mother-child dyads collected through a household survey conducted between December 2019 and January 2020. The independent variables were maternal decision-making and mental health status (general anxiety, depressive symptoms, parental stress). The dependent variable was the child’s nutritional status (thinness, stunting, underweight and overweight). Confounders were maternal income, age, and education status, and the child’s age and sex. The associations between the dependent and independent variables were determined using multivariable binary logistic regression analysis after adjusting for confounders. The adjusted odds ratios (AORs) were determined.

**Results:**

Children of mothers with mild general anxiety had lower odds of stunting than children of mothers with normal anxiety (AOR: 0.72; p = 0.034). Mothers who did not make decisions on children’s access to health care (AOR: 0.65; p < 0.001) had children with lower odds of being thin than those whose mothers made decisions on their access to health care. Children of mothers with clinically significant parenting stress levels (AOR: 0.75; p = 0.033), severe depressive symptoms (AOR: 0.70; p = 0.041) and who were not decision makers on the access of their children to health care (AOR: 0.79; p = 0.035) had lower odds of underweight.

**Conclusions:**

Maternal decision-making status and mental health status were associated with the nutritional status of children less than 6 years in a sub-urban community in Nigeria. Further studies are needed to understand how maternal mental health is associated with the nutritional status of Nigerian preschool children.

## Introduction

Mothers are the usually the primary caregivers for young children. The caregiving process can be complex and include many decisions and responsibilities around feeding a child, which is a key determinant of child malnutrition. Multiple maternal psychosocial stimuli may affect their ability to provide optimal nutritional care for their child [1–3]. One such psychosocial stimuli is depression, which is a mental health disorder.

Post-partum depression occurs in about 13% of women [4] and lead to poor parenting practices [2, 5]. It is associated with undernutrition in children because the depressed mother is unresponsive to the child, unable to form a secure attachment, and unable to provide a healthful diet for the child [6] often resulting in stunting [7]. Another psychosocial stimulus is general anxiety. Though a less consistent finding [8], chronic general anxiety may also present the same effect as depression [5, 9]. A third psychosocial stimulus is parental stress which promotes parent-centered that is counterproductive for the child’s optimal growth [10]. Though Harpham et al. [4] found no association between maternal mental health status and the child’s nutritional status in Ethiopia, an association was reported in Asia [1], suggesting possible regional variability in the impact of maternal mental health status on the child’s nutritional status.

In addition to maternal good mental health, higher levels of maternal economic status [11], more autonomous maternal decision-making ability [12] and higher maternal educational status [11, 13, 14] are factors positively associated with the nutritional status of children. Other factors associated with children’s nutritional status include the child’s sex - male children were more likely to be stunted [15]; and maternal age - children of mothers younger than 28-years had worse nutritional outcomes [16].

Although maternal psychosocial factors (general anxiety, parental stress, depressive symptoms, and empowerment status) affect children’s health behavior [17–19] and nutritional outcomes [20], there is no information on the associations between maternal related factors on the nutritional status of children in Nigeria [21, 22]. Yet, its plausible that there are associations between maternal mental health status and the nutritional status of children in Nigeria with the high prevalence of maternal mental health challenges, poor maternal decision-making power, and high prevalence of malnutrition in Nigeria. The prevalence of maternal perinatal depression ranges from 10 to 30% [23], and that of anxiety ranges from 16 to 39% [24]. In addition, 37% of children below the age of 5 years are stunted, 7% are wasted, 22% are underweight and 2% are overweight [25], with no significant changes in these figures since 2014 [26]. An estimated 2 million children under the age of five suffer from severe acute malnutrition, most of whom remain untreated [27].

Stunting, wasting and underweight are associated with increased risk for mortality in children while overweight is a risk factor for several debilitating diseases such as diabetes, heart disease, and some cancers [28, 29]. Yet, the risk of malnutrition may be lower for children whose mothers are empowered to make autonomous decisions about access to healthcare, household purchases and socialization [30–32]. This is because mothers with the ability to make independent decisions can direct household resources to the care of the children, enact their preferences in caring for and raising their children, and use their ability to socialize to increase awareness of, and access to health-promoting resources [33]. These ultimately lead to significant improvement in the health and nutritional well-being of the child [34] and reduces the odds of having children who are stunted, wasted or underweight [12, 35, 36].

There are several studies on the associations between poor maternal mental health and poor nutritional status of children [4, 37, 38]. There are, however, very few studies on the associations between maternal mental health and nutritional status of children in Nigeria [39] despite the high burden on children malnutrition in the country: Nigeria has the second highest burden of stunted children [27]. A study on the associations between maternal mental health and nutritional status of children acknowledges that there are geography and sociocultural contexts that affect the mental health [40] and the decision-making ability of mothers [41], as well as the nutritional status of children [42] and hence, the need for studies in Nigeria. Assessing the associations between maternal psychosocial status and children’s nutritional status will facilitate further studies on the pathways for these associations in the Nigeria context; and support the design of socio-cultural sensitive intervention programs to improve the psychosocial status of mothers and nutritional status of children.

In this study, we examined the associations between the decision-making and psychosocial status of mothers of children less than 6 years old in Ile-Ife Nigeria and the nutritional status of their children. We hypothesized that poor maternal autonomy (measured as lacking autonomous decision-making about health, household purchases and socialization), and poor maternal mental health status (general anxiety, parental stress, depressive symptoms) are associated with children’s poor nutritional status (thinness, stunting, underweight and overweight).

## Methods

This was a secondary analysis of cross-sectional data collected from 1549 mother-child dyads to determine the association between maternal emotional health status and early childhood caries in children less than 6 years of age resident in Ile-Ife, Nigeria. Ile-Ife is a sub-urban agrarian community located in Southwestern Nigeria with an estimated population of 501,000 people.

### Study participants, sample size and sampling procedure

Study participants for the primary study were mothers and their children aged 6 to 71 months old recruited between December 2018 and January 2019. Children present at the time of the survey, and for whom parental consent for study participation was obtained, were included in the study. The primary study excluded children with chronic medical conditions requiring prolonged use of free sugar containing medications, and those with medical conditions that increased their caries risk such as children with head and neck tumor that have undergone radiotherapy, children with Sjogren syndrome or children with HIV infection. The medical health status of the child was asked to determine their eligibility before enrolling them into the study.

The study participants were recruited using a three-level multi-stage sampling technique. The details of the sample size determination and sampling technique have been extensively described in a prior publication [43]. For this study, we computed a minimum sample size of 968 as adequate for determining the association between maternal psychosocial factors and children’s nutritional status using a malnutrition prevalence of 37% [25], error margin of 5%, and 95% confidence level. For regression, at least 300 participants were required to detect the smallest effect size based on Newsom’s method [44].

### Data collection

The dependent variable for this study was the child’s malnutrition status (underweight and overweight, stunting and thinness) while the independent variables were the maternal psychosocial status (levels of general anxiety, parental stress and depressive symptoms) and decision-making status. The confounding variables were the child’s sociodemographic variables (children’s age and sex) and the maternal sociodemographic variables (maternal age, educational status and monthly income).

#### Nutritional status

Nutritional status was determined using the World Health Organization (WHO) AnthroPlus Software, which contains the WHO Reference 2007 for 5-19-year-olds and the WHO Child Growth Standard for 0-5-year-olds. This included height, age, and weight [45]. Data on height and weight was collected in line with the International Society for the Advancement of Kinanthropometry standard protocol [46]. Children removed shoes and any heavy clothes before having their height and weight measured. Height was measured once and to the nearest 0.1 centimeter with a portable stadiometer (Seca 217). The child stood barefoot, maintained the head in a neutral position, with the neck, spinal column and knees in physiological extension and the soles of both feet and buttocks touching the vertical backboard of the stadiometer. The horizontal bar of the stadiometer was lowered until it compressed the hair to the crown [47].

Weight was measured to the nearest 0.1 kg with a portable digital scale (Generic Electronic Digital Weighing Scale). The weighing scale was zero balanced before each child stepped onto it for each measurement. The weight was measured after the digital screen fluctuations stopped and while the child was standing erect and relaxed. The weight for infants was obtained by measuring the weight of the mother carrying the child, and then subtracting the weight of the mother [47].

Children whose height for age z-scores were below minus two standard deviations (SD) from the median of the WHO reference population were considered stunted, while those with z-scores of -2.00 to 2.00 were classified as normal. Likewise, children whose weight for age z scores were below minus two SD from the reference population median were considered underweight and those with z-scores > 2.00 were classified as overweight. Children whose Body Mass Index (BMI) for age z-scores were <-2.00 SD from the reference population median were classified as thin; those aged < 5 years with z-scores > 2.00 and > 3.00 were respectively classified as overweight/obese [48].

#### General anxiety

The 7-item Generalized Anxiety Disorder-7 scale was used to measure generalized anxiety disorder [49]. The instrument had a high sensitivity and specificity. It was used in Nigeria with a group of pregnant women [50]. The Generalized Anxiety Disorder-7 score is calculated by assigning scores of 0, 1, 2, and 3, to the response categories of ‘not at all’, ‘several days’, ‘more than half the days’, and ‘nearly every day’, respectively. Scores were categorized into 0–4 = normal anxiety, 5–9 = mild anxiety, 10–14 = moderate anxiety, and 15–21 = severe anxiety [47]. The categories of moderate and severe general anxiety were combined because of the small number of participants with severe general anxiety (6 persons). The Cronbach alpha score of this scale for this study was 0.92.

#### Parenting stress

Six items from the 19-item Parenting Stress Index used in the Detroit Dental Health Project [29] were used to measure maternal stress. The Parenting Stress Index has very good to excellent internal consistency [51] and was validated for use in the Nigerian population [52]. Possible scores for each item on the index ranged from 5 = almost always, to 4 = often, 3 = sometimes, 2 = rarely, to 1 = never. Higher scores reflect more frequent experiences of stress. Scores below the 81st percentile were categorized as normal stress range; 81st to 84th percentile scores indicated borderline stress, while scores equal to and greater than 85th percentile were classified as having clinically significant levels of stress. These reference percentile levels were proposed by Abidin and Staff [53]. The Cronbach alpha score of this scale for this study was 0.89.

#### Depressive symptoms

The 20-item Centre for Epidemiologic Studies and Depression Scale, developed by Radloff [54] and validated for use in Nigeria [55], was used to determine the level of depressive symptoms. Each item in the scale was assigned scores of 0–3, depending on the frequency of symptoms per week, with the total score ranging from 0 to 60. Scores of less than 15 indicated no depressive symptoms; 15–21 mild to moderate depressive symptoms; and 21–60 major depressive symptoms. The Cronbach alpha score of this scale for this study was 0.90.

#### Decision-making status

Information on maternal decision-making ability, measured using the demographic health survey instrument [56], was based on responses to three questions: the person who usually makes decisions on her healthcare, the person who usually decides on large household purchases, and the person who usually decides on visits to family or relatives, was retrieved. The responses included: mother only, husband/ partner only, mother and husband/ partner.

### Confounding variables

Data retrieved included mother’s age at last birthday, which was categorized into three groups: ≤29 years, 30–39 years, 40 years and over. Information on mothers’ educational status (no formal education, primary school only, secondary school only or post-secondary school) and income were also retrieved. Income was defined as a monthly salary for persons in paid employment and categorized using the national Nigerian currency and wage into four categories: ≤N18,000 ($49)/month, N18,001- N30,000 ($84)/month, N30,001-N60,000 ($168)/month, and > N60,000 ($168)/month [57]. These variables were identified as confounders because of their relationship with the child’s nutritional status and maternal mental health status [58–60]. Other confounding variables were the children’s age at last birthday and the sex of the children at birth. The sociodemographic profile of the study participants has been reported in prior studies [43, 61–64].

### Standardization of examiners

Five examiners were trained to obtain measurements that were not significantly different from that of a consultant nutritionist who was considered the “gold standard.” The consistency in measurement between the trainees and the expert was assessed using inter-examiner reliability scores. Twenty children were each measured for height and weight by the expert alongside the five examiners engaged in this study. The kappa scores for stunting, underweight, overweight and normal were 0.89, 0.92, 0.95 and 0.90 respectively.

### Data Analysis

Descriptive analysis was conducted to determine the proportion of children recruited by each sociodemographic variable (age and sex), malnutrition status (underweight and overweight, stunting and thinness) and maternal decision-making status and the mental health status (levels of general anxiety, depressive symptoms, parental stress). We used multiple indictors of mental health to capture problems the individual may suffer from whether related (parental stress) or not related (general anxiety and depressive symptoms) to parenthood.

Four multivariable adjusted binary logistic regression models were developed, one for each type of malnutrition condition (underweight and overweight, stunted and thinness), with each having yes/ no responses. We used these various categories to comprehensively assess the factors associated with different types of malnutrition rather than focus on one category/ problem only in view of the extent of the problem in the country. Also, some children had more than one problem. We checked for multicollinearity in the models and dropped some variables that were correlated (r ≥ 0.5) with others from the models. We dropped off “autonomous decisions about household purchases” and “autonomous decisions about visits to family and relatives” because they were correlated with “autonomous decisions about access to health care”. Also, we dropped “maternal general anxiety” in the models that determined variables associated with underweight and thinness because it correlated with “maternal depressive symptoms” in those models. The estimated coefficients, expressed as adjusted odds ratios (AORs) and their 95% confidence intervals, were calculated. The analysis used a robust variance estimator to allow for direct estimation of the ORs. The statistical analyses were conducted with Intercooled STATA (release 15) for windows. Statistical significance was inferred at p ≤ 0.05.

### Ethics approval

Ethical approval for the study was obtained from the Obafemi Awolowo University Teaching Hospitals Complex Health Research Ethics Committee (NHREC/27/01/2009a and IRB/EC/0004553). Written informed consent was provided by the mothers and mothers on behalf of their children in the primary study.

## Results

Only 493 (31.8%) of the study participants had normal nutritional status. Others were thin (30.8%), stunted (26.8%), underweight (23.3%) and overweight/obese (11.3%). There were 68 (4.4%) mothers with moderate to severe general anxiety levels, 329 (21.2%) mothers with a clinically significant level of parenting stress and 374 (24.2%) mothers with mild to severe depressive symptoms. The majority of mothers were capable of autonomous decisions regarding access to health care (72.8%), household purchases (77.8%) and visits to family and relatives (76.6%).

Table 1 highlights the maternal mental health status and decision-making status associated with the nutritional status of the child. Maternal general anxiety and depressive symptoms were not significantly associated with the child being underweight, overweight/obese, stunted, thin or normal. The proportion of children with mothers reporting normal parental stress levels who were underweight (p = 0.009) and overweight/obese (p = 0.042) were significantly higher than mothers with borderline or clinically significant parental stress.

**Table 1:** Bivariate Analysis of the association between child-mother pairs’ characteristics and nutritional status of children younger than 6 years resident in Ile-Ife, Nigeria (N = 1549)*

Variables	Underweight n (%)	P-value	Overweight/obese n (%)	P-value	Stunted n (%)	P-value	Thin n (%))	P-value	Normal n (%)	P value
General anxiety										
Normal	292 (81.8)	0.539	137 (83.0)	0.592	331 (84.2)	0.125	357 (80.0)	0.569	386 (78.3)	0.516
Mild	52 (14.6)		20 (12.1)		47 (12.0)		65 (14.6)		85 (17.2)	
Moderate/Severe	13 (3.6)		8 (4.9)		15 (3.8)		24 (5.4)		22 (4.5)	
**Parental Stress**										
Normal	294 (82.4)	0.009	114 (69.1)	0.042	300 (76.3)	0.609	341 (76.5)	0.893	387 (78.5)	0.282
Borderline	8 (2.2)		7 (4.2)		11 (2.8)		9 (2.0)		8 (1.6)	
Clinically significant	55 (15.4)		44 (26.9)		82 (20.9)		96 (21.5)		98 (19.9)	
**Depressive symptoms**										
Normal	276 (77.3)	0.367	134 (81.2)	0.080	306 (77.9)	0.281	354 (79.4)	0.317	366 (74.2)	0.553
Mild-moderate	50 (14.0)		22 (13.3)		53 (13.5)		45 (10.1)		70 (14.2)	
Severe	31 (8.7)		9 (5.5)		34 (8.6)		47 (10.5)		57 (11.6)	
**Maternal decision-making status**										
Someone else decides on access to health care	80 (22.4)	0.132	46 (29.1)	0.727	107 (27.2)	0.513	83 (18.6)	< 0.001	152 (30.8)	0.001
Someone else decides on household purchases	79 (22.1)	0.477	40 (24.5)	0.602	95 (24.2)	0.120	73 (16.4)	0.002	112 (22.7)	0.207
Someone else decides on visits to family/relatives	77 (21.6)	0.973	41 (25.5)	0.677	105 (26.7)	0.014	72 (16.1)	< 0.001	125 (25.4)	0.016
**Age of child (mean ± sd)**	**45.3 ± 17.8**	< 0.001	**31.6 ± 19.5**	< 0.001	**40.3 ± 19.0**	0.191	**40.4 ± 17.2**	0.152	**41.7 ± 17.4**	< 0.001
0-2-year-olds	60 (16.8)	< 0.001	68 (41.2)	< 0.001	94 (23.9)	0.990	90 (20.2)	0.026	99 (20.1)	0.008
3-5-year-olds	297 (83.2)		97 (58.8)		299 (76.1)		356 (79.8)		394 (79.9)	
**Sex of child**										
Male	194 (54.3)	0.171	78 (47.3)	0.165	216 (55.0)	0.209	254 (57.0)	0.020	254 (51.5)	0.0.807
Female	163 (45.7)		87 (52.7)		177 (45.0)		192 (43.0)		239 (48.5)	
**Age of mother**										
≤ 24-years-old	28 (7.8)	0.460	13 (7.9)	0.519	32 (8.1)	0.111	30 (6.7)	0.019	48 (9.7)	0.033
25-34-years-old	204 (57.1)		91 (55.2)		225 (57.3)		294 (65.9)		271 (5.0)	
35-44-years-old	112 (31.4)		57 (34.6)		121 (30.8)		114 (25.6)		164 (33.3)	
≥ 45-years-old	13 (3.6)		4 (2.4)		15 (3.8)		8 (1.8)		10 (2.0)	
**Maternal income status**										
< N18,000 ($49) per month	110 (30.8)	0.318	34 (20.6)	0.418	103 (26.2)	0.042	104 (23.3)	0.566	145 (29.4)	0.300
N18,000 – N30,000 ($84) per month	145 (40.6)		70 (42.4)		180 (45.8)		196 (42.0)		215 (43.6)	
N30,001 – N60,000 ($168) per month	90 (25.2)		55 (33.3)		92 (23.4)		131 (29.4)		122 (24.8)	
> N60,000 per month	12 (3.4)		6 (3.6)		18 (4.6)		15 (3.3)		11 (2.2)	
**Maternal educational status**										
None	6 (1.7)	0.001	2 (1.2)	0.899	7 (1.8)	0.771	2 (0.4)	0.022	11 (2.2)	0.033
Primary	27 (7.6)		11 (6.7)		24 (6.1)		25 (5.6)		46 (9.3)	
Secondary	195 (54.6)		99 (60.0)		247 (62.9)		275 (61.7)		313 (63.5)	
Above secondary	129 (36.1)		53 (32.1)		115 (29.2)		144 (32.3)		123 (25.0)	
N	357 (100.0)		165 (100.0)		393 (100.0)		446 (100.0)		493 (100.0)	

*Some children had more than one nutritional status

In addition, maternal decision-making status was associated with being stunted, thin and normal nutritional status. A higher proportion of children with mothers able to make decisions on visits to family/relatives were stunted compared to children of mothers unable to make this decision autonomously (p = 0.014). A higher proportion of children with mothers able to decide on access to health care (p < 0.001), household purchase (p = 0.002) and visits to family/relatives (p < 0.001) were thin compared to children of mothers unable to make those decisions autonomously. Also, a higher proportion of children with mothers who can make decisions about access to health care (p = 0.001), and visits to family/relatives (p = 0.016) had normal weight compared with children of mothers who could not make those decisions autonomously.

Table 2 shows the outcome of the multivariable logistic regression analysis, determining the association between maternal mental health and decision-making status and children’s nutritional status.

**Table 2:** Multivariable logistic regression analysis of maternal psychosocial and economic factors associated with malnutrition among children younger than 6 years resident in Ile-Ife, Nigeria (N = 1549)

Variables	n (%)	Underweight AOR (95% CI)	p-value	Overweight/obese AOR (95% CI)	p-value	Stunted AOR (95% CI)	p-value	Thin AOR (95% CI)	p-value
Maternal General Anxiety									
Normal	1,238 (79.9)			1	-	1	-		
Mild:	243 (15.7)			0.79 (0.46—1.37)	0.397	0.72 (0.53—0.98)	0.034		
Moderate/Severe	68 (4.4)			1.01 (0.45—2.27)	0.989	0.81 (0.51—1.28)	0.366		
**Maternal Parenting Stress**									
Normal	1,184 (76.4)	1.00	-	1.00	-	1.00	-	1.00	-
Borderline	36 (2.3)	0.93 (0.50—1.71)	0.814	2.10 (0.96—4.56)	0.062	1.31 (0.81—2.12)	0.279	0.80 (0.45—1.42)	0.437
Clinically significant	329 (21.2)	0.75 (0.58—0.98)	0.033	1.15 (0.78—1.69)	0.484	0.98 (0.78—1.23)	0.884	0.92 (0.76—1.12)	0.391
**Maternal Depressive Symptoms**									
Normal	1,175 (75.8)	1.00	-	1.00	-	1.00	-	1.00	-
Mild-moderate	207 (13.4)	0.91 (0.70—1.19)	0.503	1.20 (0.71—2.01)	0.496	1.22 (0.93—1.59)	0.150	1.81 (0.62—1.06)	0.128
Severe	167 (10.8)	0.70 (0.50—0.98)	0.041	0.54 (0.25—1.18)	0.122	0.95 (0.67—1.34)	0.753	1.00 (0.78—1.28)	0.984
**Maternal Decision-Making- Status**									
Someone else does not decide on access to health care		1.00	-	1.00	-	1.00	-	1.00	-
Someone else decides on access to health care	394 (27.2)	0.79 (0.63—0.98)	0.035	1.12 (0.79—1.60)	0.528	1.06 (0.87—1.28)	0.583	0.65 (0.53—0.80)	< 0.001
**Age of Child**									
		1.00	-	1.00	-	1.00	-	1.00	-
Age (months)		1.02 (1.01—1.02)	0.045	0.98 (0.97—0.98)	< 0.001	1.00 (1.00—1.01)	0.565	1.00 (1.00—1.01)	0.097
**Sex of Child**									
Male	792 (51.1)	1.00	-	1.00	-	1.00	-	1.00	-
Female	757 (48.9)	0.95 (0.79—1.13)	0.547	1.10 (0.80—1.51)	0.558	0.89 (0.75—1.06)	0.198	0.84 (0.72—0.98)	0.029
**Age of Mother**									
≤ 24-years-old	129 (8.3)	1.00	-	1.00	-	1.00	-	1.00	-
25-34-years-old	919 (59.3)	0.90 (0.63—1.27)	0.555	0.98 (0.54—1.79)	0.954	0.95 (0.69—1.30)	0.732	1.22 (0.88—1.70)	0.231
35-44-years-old	459 (29.6)	0.92 (0.63—1.33)	0.653	1.35 (0.71—2.54)	0.358	1.04 (0.74—1.47)	0.825	0.93 (0.65—1.33)	0.687
≥ 45-years-old	42 (2.7)	1.10 (0.62—1.95)	0.743	1.40 (0.44—4.43)	0.565	1.68 (1.02—2.78)	0.041	0.79 (0.39—1.61)	0.517
**Maternal Educational Status**									
None	23 (1.5)	1.00	-	1.00	-	1.00	-	1.00	-
Primary:	115 (7.4)	0.89 (0.41—1.89)	0.754	0.95 (0.21—4.43)	0.952	0.65 (0.33—1.31)	0.229	2.85 (0.76—10.68)	0.120
Secondary:	977 (63.1)	0.86 (0.42—1.74)	0.674	0.87 (0.21—3.58)	0.846	0.78 (0.42—1.43)	0.419	3.23 (0.90—11.62)	0.072
Above secondary:	434 (28.0)	1.18 (0.58—2.41)	0.652	1.16 (0.28—4.88)	0.838	0.78 (0.42—1.47)	0.443	3.49 (0.97—12.60)	0.056
**Maternal Income Status**									
< N18,000 per month:	422 (27.2)	1.00	-	1.00	-	1.00	-	1.00	-
N18,000 – N30,000 per month	665 (42.9)	0.79 (0.63—0.99)	0.039	1.05 (0.68—1.62)	0.813	1.00 (0.80—1.23)	0.964	1.04 (0.84—1.27)	0.738
N30,001 – N60,000 per month	417 (26.9)	0.75 (0.58—0.97)	0.028	1.16 (0.72—1.86)	0.549	0.79 (0.61—1.04)	0.091	1.09 (0.87—1.37)	0.474
> N60,000 per month	45 (2.9)	0.79 (0.48—1.33)	0.378	1.18 (0.48—2.94)	0.718	1.40 (0.92—2.13)	0.114	1.11 (0.70—1.77)	0.645
Constant		0.17 (0.08—0.38)	< 0.001	0.23 (0.05—1.05)	0.057	0.37 (0.18—0.74)	0.005	0.09 (0.02—0.32)	< 0.001
*Pseudo R* ^*2*^		*0.04*		*0.05*		*0.01*		*0.02*	

### Variables associated with children being underweight

Mothers with clinically significant parenting stress levels were less likely to have children who were underweight (AOR 0.75; 95% CI: 0.58—0.98; p = 0.033) than children of mothers who had normal parenting stress. Also, children of mothers who had severe depressive symptoms were less likely to be underweight than children of mothers with normal depressive symptoms (AOR 0.70; 95% CI: 0.50—0.98; p = 0.041). Mothers with a substitute decision maker on access to health care were less likely to have children who were underweight compared with children of mothers who made autonomous decisions on access to health care (AOR 0.79; 95% CI: 0.63—0.98; p = 0.035).

### Variables associated with children being overweight/obese

There was no independent variable associated with overweight/obese.

### Variables associated with children being stunted

The only maternal explanatory variable associated with children being stunted was general anxiety. Mothers with mild general anxiety were less likely to have children who were stunted than mothers with normal general anxiety (AOR 0.72; 95% CI: 0.53, 0.98; p = 0.034).

### Variables associated with children being thin

The only maternal explanatory variable associated with children being thin was maternal decision-making status. Mothers with someone else making their health access decision were less likely to have children who were thin than those who made the decisions themselves (AOR: 0.65; 95% CI: 0.53—0.80; p < 0.001).

## Discussion

The study results suggest a complex relationship between maternal psychosocial factors and the nutritional status of children. The maternal variables associated with child malnutrition in this study population were mild general anxiety, clinically significant parenting stress and severe depressive symptoms. Mothers with mild general anxiety seem less likely to have children who were stunted; while mothers with severe depressive symptoms and clinically significant parenting stress seem less likely to have underweight children. Similarly, mothers for whom someone else decides on access to health care were less likely to have children who were underweight and thin. Our study hypotheses were therefore supported by the study findings although the directions of the associations raise more questions than they provide answers.

One of this study strengths was the inclusion of multiple potential risk factors in the study model, thereby increasing the possibility of simulating real-life risk exposure. However, the low R^2^ of the models studied indicate that variables in the models did not account for most of the variations in children’s nutritional status. Some confounding and mediating factors - the child’s birth weight and household food insecurity, child’s physical health, breastfeeding status [12] and parental Body Mass Index (BMI) [65] - were not included in the regression analysis as these were not collected in the primary dataset. Childhood illness is also a risk factor for growth failure [66] and was not included. The primary study excluded children who were on prolonged use of sweetened medication, which implied the exclusion of children with chronic illnesses; this exclusion helped reduce some confounders for this study. Also, this was a cross sectional study and so we are unable to determine a causal relationship between the variables. In addition, the data excluded details of children younger than 6 months old since the primary data was collected for children who should have erupted teeth. Our study findings are therefore not applicable to neonates and young infants. Despite these limitations, this study was able to demonstrate that some maternal psychosocial factors played a significant role in children’s nutritional status in the study setting.

Interestingly, mothers with severe depressive symptoms and clinically significant parenting stress seem less likely to have children who were underweight. We postulate that in the study environment, mothers derive some form of social capital from their social networks and ties to other individuals, groups, and the larger community [67, 68]. For a community-based society like Nigeria where communal living and access to community support is high [69], individuals with mental health problems have increased social support which may improve the health and wellbeing of the child [70]. In Nigeria, where the number of trained mental health professionals is few and access to social service is poor, families often play a prominent role in managing mental health disorders [71]. These social ties may ameliorate the impact of maternal mental health problems on the nutritional health of the child. Prior studies conducted in developing countries reported an association between maternal depressive symptoms and the child being underweight [72–75]. No association was found between these two variables in Brazil [76]. Other studies also found an association between maternal depression and the child being stunted [72, 73], which we did not find in our study. Future studies are needed to explore and identify if there are cultural nuances that moderate or mediate the association between maternal depression and nutritional status of children.

The study also showed that maternal decision-making ability about access to health care affected children’s nutritional status negatively: having someone else – likely the husband in a patriarchal society like Nigeria [77] - take decisions about healthcare access reduces the risk of being underweight and thin. These findings may reflect a societal context wherein male spouses are often better educated about health issues and wealthier and are better placed to make the needed out-of-pocket expense for health care [78, 79]. In effect, women may be able to facilitate access of the child to health care when they receive funds from significant others.

On the contrary, women who can make independent decisions about access to health care, are likely to be working mothers. Health care is often paid for through out-of-pocket expenses in Nigeria, and thus, managing childcare health needs require some level of financial independence [79]. Working mothers, however, have less time to pay attention to the nutritional needs of their young children since they resume back to work shortly after the birth of the child [80–83]. Paid maternity leave can improve children’s nutritional health and is an important s policy tool because it may enable many maternal practices that can improve the nutritional status of the child [84, 85]. The study findings and our postulations need to be further tested, and reasons proffered for the association between the reduced odds for wasting when mothers can make autonomous decisions about a visit to family/relatives.

We also observed that mild general anxiety was protective from stunting. Mild anxiety may enable mothers to react more sensitively to the health needs of their children including the need for nutritional care. A prior study had demonstrated that maternal anxiety was associated with child obesity [86]. This may be because anxiety is associated with maternal overstimulation [87], which then results in forceful feeding of the child [88]. High anxiety level on the contrary, may be counterproductive and result in low diet quality for the child [89].

Our findings have contributed to highlighting the complexity of the interactions between maternal psychosocial factors, children’s nutritional status and the country of origin of the study. When we compared our findings with those of other studies, we observed disparities in findings for different countries. This points to the likelihood of social context being a possible mediating factor in the relationship between maternal psychosocial factors and children’s nutritional status. If confirmed, countries will need to understand how these factors are associated, and how other factors mediate or moderate their relationships.

For Nigeria, the findings indicate that the level of general anxiety, parenting stress, depressive symptoms, and decision-making status of mothers should be given due consideration in the design and implementation of nutrition programs for children younger than 6 years. Broadening obesity prevention efforts to include a reduction in maternal depression burden may be an important nutrition implementation guideline in the study setting [90]. Also, a perinatal assessment for depression and general anxiety status may likely reduce the risk for children’s growth failure. Access to counselling interventions that draw on techniques from cognitive-behavioral therapy and problem-solving therapy, provided within a supportive group can help rebuild a sense of agency by promoting self-efficacy for mothers with psychosocial challenges [91].

## Conclusion

Mothers in Ile-Ife, Nigeria with mild general anxiety, clinically significant parenting stress, severe depressive symptoms, and less decision-making autonomy regarding access to healthcare, seem less likely to have children younger than 6 years with a form of malnutrition. These results seem counterintuitive though the socio-cultural context of the study location may explain the findings. The study results seem to suggest that relationships between maternal psychosocial status and children’s nutritional status may be moderated by sociocultural context. Further studies are needed to understand how sociocultural factors may moderate maternal psychosocial status and children’s nutritional status as this can inform the design of malnutrition programs for children.

## Data Availability

All data generated for this study are presented in the manuscript. The dataset for the online study data can however be accessible on reasonable request from one the study author, Morenike Oluwatoyin Folayan, toyinukpong@yahoo.co.uk.

## Abbreviations

AOR: Adjusted Odds Ratio
BMI: Body Mass Index
CI: Confidence Interval
SD: Standard Deviation
WHO: World Health Organization
